# Genetic Variant rs10757278 on Chromosome 9p21 Contributes to Myocardial Infarction Susceptibility

**DOI:** 10.3390/ijms160511678

**Published:** 2015-05-21

**Authors:** Guangyuan Chen, Xiuhua Fu, Guangyu Wang, Guiyou Liu, Xiuping Bai

**Affiliations:** 1 The First Hospital of Harbin, Harbin 150070, Heilongjiang, China; E-Mail: mengyao0451@163.com; 2 The Department of Internal Circulation, the Second People’s Hospital of Mudanjiang, Mudanjiang 157013, Heilongjiang, China; E-Mail: hailepan0451@163.com; 3 Department of Gastrointestinal Medical Oncology, the Affiliated Tumor Hospital of Harbin Medical University, Harbin 150081, Heilongjiang, China; E-Mail: guangyuwanghrb@163.com; 4 Genome Analysis Laboratory, Tianjin Institute of Industrial Biotechnology, Chinese Academy of Sciences, Tianjin 300308, China; E-Mail: liuguiyou1981@163.com; 5 Department of Cardiology, the Fourth Affiliated Hospital of Harbin Medical University, Harbin 150001, Heilongjiang, China

**Keywords:** myocardial infarction, rs10757278, meta-analysis

## Abstract

Large-scale genome-wide association studies (GWAS) have revealed that rs10757278 polymorphism (or its proxy rs1333049) on chromosome 9p21 is associated with myocardial infarction (MI) susceptibility in individuals of Caucasian ancestry. Following studies in other populations investigated this association. However, some of these studies reported weak or no significant association. Here, we reevaluated this association using large-scale samples by searching PubMed and Google Scholar databases. Our results showed significant association between rs10757278 polymorphism and MI with *p* = 6.09 × 10^−22^, odds ratio (OR) = 1.29, 95% confidence interval (CI) 1.22–1.36 in pooled population. We further performed a subgroup analysis, and found significant association between rs10757278 polymorphism and MI in Asian and Caucasian populations. We identified that the association between rs10757278 polymorphism and MI did not vary substantially by excluding any one study. However, the heterogeneity among the selected studies varies substantially by excluding the study from the Pakistan population. We found even more significant association between rs10757278 polymorphism and MI in pooled population, *p* = 3.55 × 10^−53^, after excluding the study from the Pakistan population. In summary, previous studies reported weak or no significant association between rs10757278 polymorphism and MI. Interestingly, our analysis suggests that rs10757278 polymorphism is significantly associated with MI susceptibility by analyzing large-scale samples.

## 1. Introduction

Myocardial infarction (MI) is a complex human disease with a strong genetic component [1]. MI is heritable and among the leading causes of death and disability worldwide [2]. Most of the MI cases occur in individuals >65 years old, 5%–10% of new MI cases occur in younger patients and these events are associated with substantially greater heritability [2]. Genome-wide association studies (GWAS) are considered to be new and powerful approaches to detect the genetic variants of human complex diseases. Large-scale GWAS have been conducted and reported common single nucleotide polymorphisms (SNPs) on chromosome 9p21.3for MI and coronary artery disease in European ancestry [2,3].

Helgadottir *et al.* investigated a total of 4587 MI cases and 12,767 controls [3]. They identified variant rs10757278 on chromosome 9p21, adjacent to the tumor suppressor genes CDKN2A and CDKN2B, was associated with MI with high significance (*p* = 1.00 × 10^−20^, odds ratio (OR) = 1.28, 95% confidence interval (CI) 1.22–1.35) [3].

GWAS and candidate gene studies also investigated the association between rs10757278 polymorphism and MI in other populations. Some studies reported significant association between rs10757278 polymorphism and MI [4,5,6,7,8]. However, other studies reported a weak or negligible association between rs10757278 polymorphism and MI [9,10,11,12,13,14]. Meta-analysis method involves combining and analyzing quantitative evidence from related studies to produce results based on a whole body of research [15]. Considering the important role of rs10757278 polymorphism in MI risk and inconsistent results reported by previous studies, we reevaluated this association using a meta-analysis method by searching the PubMed (http://www.ncbi.nlm.nih.gov/pubmed) and Google Scholar databases (http://scholar.google.com/).

## 2. Results

### 2.1. Literature Search

**Figure 1:**
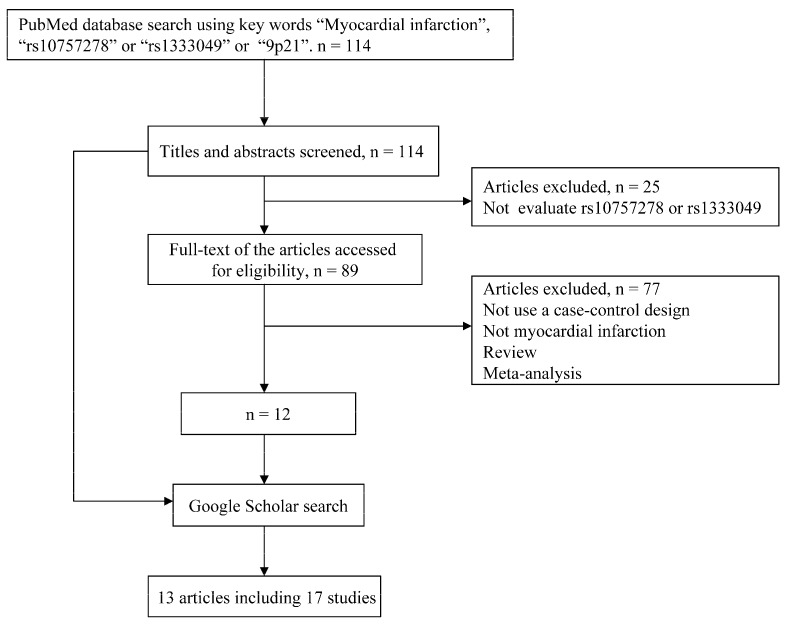
Flow chart of meta-analysis for exclusion or inclusion of individual articles.

A total of 114 articles were identified through PubMed database and 17 independent studies were finally included for following analysis. More detailed information about the inclusion or exclusion of selected studies was described in Figure 1. The main characteristics of the included studies are described in Table 1.

**Table 1:** The selected studies investigating the association between rs10757278 and Myocardial infarction (MI).

Study	SNP/Risk Allele	Country	Ethnicity	Case #	Control #	Quality Score	Genotyping Platform
[4]	rs10757278/A	China	Asian	432	430	8	GenomeLab SNPstream
[9]	rs1333049/C	China	Asian	425	1377	8	TaqMan
[16]	rs1333049/C	China	Asian	142	192	8	PCR
[17]	rs1333049/C	China	Asian	520	560	8	NA
[18]	rs10757278/C	China	Asian	1515	5019	8	NA
[5]	rs1333049/C	Japan	Asian	589	2475	9	MALDI-TOF MS
[10]	rs10757278/A	India	Asian	87	150	8	PCR
[6]	rs1333049/C	Pakistan	Asian	2587	2573	8	NA
[11]	rs10757278/A	Russia	Siberian	197	417	8	NA
[12]	rs10757278/G	Italy	Caucasian	416	308	8	ABI PRISM 7900HT
[7]	rs10757278/G	Germany	Caucasian	3657	1211	9	TaqMan
[3]	rs10757278/G	Iceland (discovery)	Caucasian	1067	6728	9	IlluminaHap300
[3]	rs10757278/G	Iceland (replication)	Caucasian	665	3533	9	IlluminaHap300
[3]	rs10757278/G	United States (Atlanta)	Caucasian	596	1284	9	IlluminaHap300
[3]	rs10757278/G	United States (Philadelphia)	Caucasian	582	504	9	IlluminaHap300
[3]	rs10757278/G	United States (Durham)	Caucasian	1137	718	9	IlluminaHap300
[8]	rs10757278/G	United States	Caucasian	310	560	9	TaqMan
				*n* = 14,924	*n* = 28,039		

The Quality Score of included studies were scored based on the criteria developed by Clark *et al*. [19] to evaluate the quality of genetic association studies. #, the number of case and control samples; NA, Genotyping platform is not available.

### 2.2. Heterogeneity Test and Meta-Analysis

**Figure 2:**
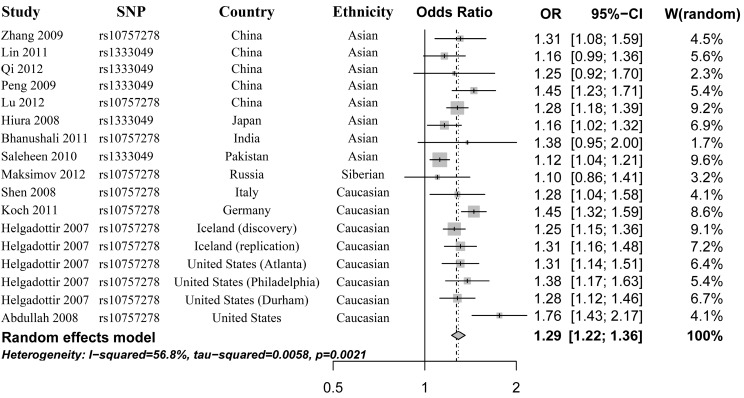
Forest plot for the meta-analysis of rs10757278 polymorphism using an additive model. The risk alleles are G for rs10757278 polymorphism and C for rs1333049 polymorphism. The additive genetic model (allele model) for this meta-analysis can be described as G allele *versus* A allele for rs10757278, and C allele *versus* G allele for rs1333049. W, weight.

We first evaluated the genetic heterogeneity of rs10757278 polymorphism among the selected studies using additive model. We observed significant heterogeneity with *p* = 0.0021 and *I*^2^ = 56.8%. We calculated the overall OR by the random-effect model. Our results showed significant association between rs10757278 polymorphism and MI with *p* = 6.09 × 10^−22^, OR = 1.29, 95% CI 1.22–1.36 (Figure 2).

### 2.3. Heterogeneity Test and Subgroup Analysis

We further performed a subgroup analysis in Asian and Caucasian populations. We did not identify significant heterogeneity in Asian (*p* = 0.0848 and *I*^2^ = 44.1%) and Caucasian population (*p* = 0.1354 and *I*^2^ = 46%).However, we observed moderate heterogeneity (*I*^2^ = 25%–50%). We found significant association between rs10757278 polymorphism and MI in Asian population with *p* = 1.82× 10^−17^, OR = 1.21, 95% CI 1.16–1.27 and Caucasian population with *p* = 8.51× 10^−39^, OR = 1.34, 95% CI 1.28–1.40.

### 2.4. Sensitivity Analysis

By excluding any one study, we identified that the association between rs10757278 polymorphism and MI did not vary substantially. By excluding the study from the Pakistan population, we observed no heterogeneity in pooled population (*p* = 0.0733 and *I*^2^ = 36.3%) and Asian population (*p* = 0.4283 and *I*^2^ = 0%). We found significant association between rs10757278 polymorphism and MI in Asian population with *p* = 6.22 × 10^−17^, OR = 1.26, 95% CI 1.20–1.34 and pooled population with *p* = 3.55 × 10^−53^, OR = 1.31, 95% CI 1.26–1.35.

### 2.5. Publication Bias Analysis

**Figure 3:**
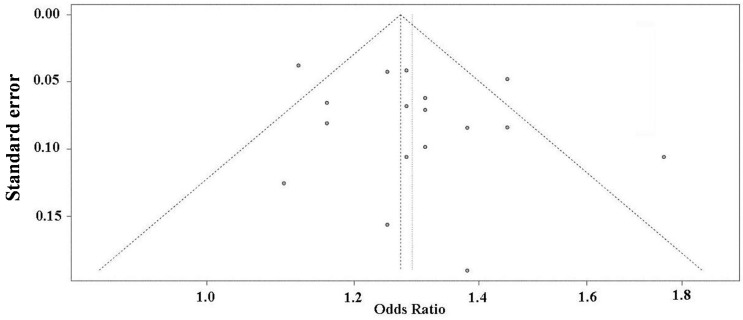
Funnel plot for publication bias analysis of the selected studies investigating the association between rs10757278 polymorphism and MI. The *X*-axis stands for the ORs and the *Y*-axis is the standard error for each of the selected studies. A linear regression based approach proposed by Egger *et al.* [19] is used to evaluate the asymmetry of the funnel plot.

The funnel plot is a symmetrical inverted funnel (Figure 3). The linear regression test suggests no significant publication bias with *p* = 0.263.

## 3. Discussion

Large-scale GWAS reported the association between rs10757278 variant and its proxy rs1333049 (rs10757278 and rs1333049 are practically equivalent, with linkage disequilibrium (LD) *r*^2^ = 1 in HapMap CEU populations) and MI [2,3]. GWAS and candidate gene studies also investigated the association between rs10757278 polymorphism and MI in other populations. However, some of these studies reported a weak or negligible association between rs10757278 polymorphism and MI [9,10,11,12,13,14].

The novelty and significance of this study can be described as the following. First, we conducted a careful literature search in PubMed and Google Scholar databases. We reevaluated this association using the relatively large-scale samples (*n* = 42,963). By careful quality evaluation, data extraction, heterogeneity test, meta-analysis, sensitivity analysis, and publication bias analysis, we observed significant association between rs10757278 polymorphism and MI with *p* = 6.09 × 10^−22^, OR = 1.29, 95% CI 1.22–1.36.

Second, we further performed a subgroup analysis in Asian and Caucasian populations. We observed moderate heterogeneity (*I*^2^ = 25%–50%). We found significant association between rs10757278 polymorphism and MI in Asian population and Caucasian population. We identified that the association between rs10757278 polymorphism and MI did not vary substantially by excluding any one study. However, the heterogeneity among the selected studies varies substantially by excluding the study from the Pakistan population. We found even more significant association between rs10757278 polymorphism and MI in pooled population with *p* = 3.55 × 10^−53^ after excluding the study from Pakistan population.

Third, prior to our submission (27 April 2015), we accessed the PubMed database. We did not find any study investigating the association between the rs10757278 polymorphism and MI by a meta-analysis method. To our knowledge, this is the first meta-analysis that further supports the association between rs10757278 polymorphism and MI susceptibility.

Szpakowicz *et al.* performed a retrospective analysis of data collected prospectively in two independent registries of consecutive patients to investigate the association of the 9p21.3 locus (rs10757278, rs1333049 and rs4977574 polymorphisms) with five-year overall mortality in patients with ST-elevation myocardial infarction [20]. They found that 9p21.3 locus is associated with five-year survival in high-risk patients with myocardial infarction [20]. Zeng *et al.* investigated whether rs10757278 was associated with acute coronary syndrome (ACS) in a Chinese Han population [21]. They performed a case-control analysis using 359 ACS patients and 398 controls [21]. They found that rs10757278 GG genotype was associated with a significantly elevated risk of ACS, and was significantly associated with recurrent angina compared with the AA and AG genotypes [21].

It is recognized that the human chromosome 9p21 is a risk factor for a first coronary heart disease (CHD) event. Until now, it is unclear about the association of 9p21 with risk of subsequent events in patients with established CHD. Patel *et al.* performed a systematic review and meta-analysis of the association between genetic variants at chromosome 9p21 and risk of first *versus* subsequent CHD events [22]. They calculated the power to detect an association of 9p21 variants with subsequent CHD events using a minor allele frequency (MAF) of rs10757278 polymorphism of 50% [22]. Their results showed that 9p21 had differential association with risk of first *versus* subsequent CHD events [22]. The 9p21 was associated with a pooled hazard ratio (HR) of a first event of 1.19 and subsequent events of 1.01 per risk allele [22]. In established CHD individuals, 4436 subsequent events indicated about 99% and 91% power to detect a per-allele HR of 1.19 or 1.10, respectively [22].

Despite these interesting results, we also realized a limitation in this study. Here, we investigated the association between rs10757278 and MI with additive model. It is reported that most meta-analyses used an additive genetic model [23]. In general, this model performs well when the true underlying genetic model is uncertain [23]. It was also important to analyze the association using dominant model and recessive model [24]. However, the dominant and recessive models required exact genotype numbers of all studies. Future studies using genotype data are required to replicate these findings.

## 4. Methods and Materials

### 4.1. Literature Search

We searched PubMed and Google Scholar databases to select all possible studies with key words “Myocardial infarction”, “rs10757278”or “rs1333049” or “9p21”. The literature search was updated on 17 December 2014.

### 4.2. Inclusion Criteria

The selected studies must (1) use a case-control design; (2) evaluate the association between rs10757278 (or its proxy rs1333049) polymorphism and MI; (3) provide an OR with 95% CI for allele model; or (4) provide sufficient data to calculate the OR and 95% CI for allele model; and (5) rs10757278 (or its proxy rs1333049) polymorphism must be in Hardy-Weinberg equilibrium (HWE).

### 4.3. Quality Evaluation

The quality evaluation criteria proposed by Clark *et al.* were selected to evaluate the quality of selected studies [19]. This scoring system included ten components. A component is scored as 1 if it is present or 0 if it is absent. We got a scoring range of 0–10 for each of the selected studies [19]. These studies were scored as ‘‘good’’ if the score was greater than or equal to 8, ‘‘mediocre’’ if the score was 5–7 and ‘‘poor’’ if the score was less than 4 [25].

### 4.4. Data Extraction

For all the selected studies, we extracted (1) the name of the first author; (2) the year of publication; (3) the population and ethnicity; (4) the numbers of MI cases and controls; (5) the genotyping platform; (6) the OR with 95% CI or to calculate the OR and 95% CI; and (7) the quality score.

### 4.5. Genetic Model

Using the LD information from the 1000 Genomes Project in HaploReg (Version 2) [26], we identify that rs10757278 and rs1333049 are practically equivalent, with LD (*r*^2^) = 0.98, LD (*D*’) = 0.99 in EUR (European) population, LD (*r*^2^) = 0.94, LD (*D*’) = 0.98 in ASN (East Asian) population, and LD (*r*^2^) = 0.97, LD (*D*’) = 0.99 in AMR (Ad Mixed American) population. The rs10757278 polymorphism includes A and G alleles, among which A is the reference allele and G is the variant allele. The rs1333049 polymorphism includes G and C alleles, among which G is the reference allele and C is the variant allele. The frequencies of the rs10757278 (A) and rs1333049 (C) are also almost equivalent (Table 2). We selected the additive genetic model (allele model) for further meta-analysis, which can be described as G allele *versus* A allele for rs10757278, and C allele *versus* G allele for rs1333049 [24].

**Table 2:** The selected studies investigating the association between rs10757278 and MI.

Chromosome	Position (hg19)	Variant	Reference Allele	Altered Allele	AMR Freq.	ASN Freq.	EUR Freq.
9	22124477	rs10757278	A	G	0.5	0.51	0.48
9	22125503	rs1333049	G	C	0.5	0.5	0.47

AMR, Ad Mixed American; ASN, East Asian; EUR, European; Freq., frequency.

### 4.6. Heterogeneity Test

Genetic heterogeneity among the selected studies is evaluated using Cochran’s *Q* test and $I^{2}=\frac{\left(Q-(k-1)\right)}{Q}\times 100\%$ statistic. Cochran’s *Q* test approximately follows a χ^2^ distribution with *k*−1 degrees of freedom (*k* stands for the number of studies for analysis). *I*^2^ is a measure of heterogeneity and a statistic that indicates the percentage of variance in a meta-analysis that is attributable to study heterogeneity [27]. Low, moderate, large and extreme heterogeneity corresponded to 0%–25%, 25%–50%, 50%–75% and 75%–100% [28]. A *p* < 0.01 from Cochran’s *Q* test and *I*^2^ > 50% were considered to be statistically significant heterogeneity.

### 4.7. Meta-Analysis

If there is no significant heterogeneity among the included studies, the pooled OR is calculated by the fixed effect model (Mantel-Haenszel), otherwise the OR is calculated by random-effect model (Der Simonian-Laird). *Z* test is used to determine the significance of OR. All statistical tests for heterogeneity and meta-analysis were computed using *R* Package (http://cran.r-project.org/web/packages/meta/index.html; R: http://www.r-project.org/).

### 4.8. Sensitivity Analysis

We omit each study, one at a time, to assess the influence of each individual study on the pooled OR and 95% CI as well as the association between rs10757278 and MI.

### 4.9. Publication Bias Analysis

A funnel plot from Egger *et al.* is used to investigate potential publication bias [29,30]. Meanwhile, a linear regression based approach, proposed by Egger *et al.*, is used to test for publication bias, which evaluate the asymmetry of the funnel plot to provide statistical evidence, with a *p* < 0.01 indicating that there was a significant publication bias [31].

## 5. Conclusions

Previous studies reported weak or no significant association between rs10757278 polymorphism and MI. Our analysis suggests that rs10757278 polymorphism is significantly associated with MI susceptibility by analyzing large-scale samples.
